# Patient Socioeconomic and Cultural Factors Associated With Fidelity to Guideline-Concordant Breast Cancer Therapy Delivery in Botswana

**DOI:** 10.1200/GO.22.15000

**Published:** 2022-05-05

**Authors:** Lebogang T. Mokokwe, Modesty Obasohan, Tlotlo Ralefala, Frances Barg, Mosepele Mosepele, Robert Gross, Yehoda M. Martei

**Affiliations:** ^1^ Botswana—University of Pennsylvania Partnership, Gaborone, Botswana; ^2^ University of Pennsylvania, Philadelphia, PA; ^3^ Princess Marina Hospital, Gaborone, Botswana; ^4^ University of Botswana, Gaborone, Botswana

## Abstract

Breast cancer patients, including patients living with HIV (PLWH), in sub-Saharan Africa experience a disproportionate burden of mortality. Adherence to treatment guidelines improves quality of care and survival outcomes. We therefore sought to study patient socioeconomic and cultural factors associated with treatment fidelity and how this may differ for PLWH in Botswana.

**METHODS:**

This was a qualitative study design with deviance sampling of high and low fidelity patients who initiated treatment for stage I-III breast cancer. One-on-one phone interviews were conducted using semi-structured interviews, guided by the Theory of Planned Behavior. The final sample size was determined by thematic saturation. Transcribed interviews were double-coded and analyzed in NVivo using an integrated analysis approach. We maintained a kappa statistic of 0.8 between coders.

**RESULTS:**

Fifteen high and 15 low fidelity patients were enrolled from August 25-December 15, 2020. Ten out of 30 of the cohort were PLWH. Barriers to treatment adherence included concerns about safety and efficacy of chemotherapy, lack of trust in the care team, lack of psychosocial support, financial toxicity, geographical inaccessibility, and other health-system barriers. Drivers and manifestations of stigma, including intersectional stigma of cancer fatalism in PLWH were identified as prominent barriers. Conversely, self-acceptance and de-stigmatization were identified as facilitators of treatment initiation. Additional facilitators included knowledge of curative intent, anticipated management of side effects, self-motivation, social support, and peer support. COVID-19 pandemic amplified existing socioeconomic barriers especially for patients with food insecurity and geographic inaccessibility.

**CONCLUSION:**

The study identified multi-level modifiable patient and related health-system factors associated with treatment initiation and adherence. PLWH experienced unique barriers including intersectional stigma, which is a critical finding and warrants further evaluation. The facilitators in this study provide opportunities for leveraging existing strengths within the specific context to design multilevel implementation strategies to increase treatment fidelity of guideline concordant breast cancer therapy.

